# On Modern Progress in Ophthalmic Medicine and Surgery

**Published:** 1894-03-10

**Authors:** Robert Brudenell Carter

**Affiliations:** Consulting Ophthalmic Surgeon to St. George's Hospital


### On Modern Progress in Ophthalmic Medicine and Surgery.

By Robert Brudenell Carter, F.R.C.S., Consulting Ophthalmic Surgeon to St. George's Hospital.

I selected the history of the successful treatment of glaucoma, as the first subject to be dealt with in these papers, because it served to illustrate an advance which was not merely relative, but absolute. A disease previously incurable, and always terminating in blindness, has been rendered completely amenable to surgical control, subject only to the condition that the required operation be performed in time. The history of improvements in the operations for cataract is of scarcely inferior interest; but it has to deal with improvements only, and not with the positive conquest of a new domain. Nevertheless, many of the improvements have been radical, and their aggregate effect has been to diminish, probably by one-half, the number of persons who, at any given time, are deprived of useful vision by a change which is of very frequent occurrence in advanced life, and which cannot be called uncommon at any period of existence.

As a matter of nomenclature, the word cataract, like the word glaucoma, is a survival. Glaucoma was originally applied to denote, not hardness of the eyeball, but the presence of a greenish reflection from the pupil, by which the later stages of hardness were often accompanied. In advanced "cataract" the pupil assumes a whitish aspect; and the word gave expression to the hypothesis that some kind of veil or curtain had fallen over and obscured it. The knowledge that ordinary cataract was due to opacity of the naturally transparent crystalline lens of the eye, led after a time to a division of cataracts into the "true" and the "false," the former being due to the presence of an opaque lens, the latter to the occlusion of the pupil by fibrous or membranous veils by which the lens had been replaced. After a time the adjectives passed out of use, the word cataract became restricted to lenticular opacities, and capsular or other films were described in language expressive of their nature.

The first great advantage afforded by the ophthalmoscope in relation to cataract was certainty of diagnosis, In former times it was difficult to distinguish between opacity of the lens and an appearance of discolouration, insomuch that the points of differential diagnosis between "cataract" and "glaucoma" were in many text-books printed in parallel columns, besides being based upon foundations which we now know to have been untrustworthy. In the eyes of persons in the decline of life the pupils are seldom of the clear bright black of youth, the change being produced partly by an actual alteration in the colour of the lens substance, which, although as bright as a diamond in childhood, gradually becomes of an amber tint, and partly by the modified quality of the light reflected from the back of the eye, in consequence of changes of pigmentation arising either from disease or from senility. It would be waste of time to dwell upon the "catoptric" and other tests which were employed in the diagnosis of cataract, most of them being fallacious, and all of them incomplete; and it is sufficient to say that the reflected light of the ophthalmoscopic mirror, by displaying the absolute lines of any opacity which may exist, has rendered the diagnosis a matter of ease and certainty, no longer resting upon the possibly erroneous interpretation of doubtful appearances, but upon visible fact. I have no doubt that, in the pre-ophthalmoscopic days, numbers of persons were operated upon for cataract who were not the subjects of that affection, and whose loss of sight was due to one out of several causes of a totally different character. Of almost equal value with the ophthalmoscopic examination is that by "focal illumination," in which a pencil of convergent light is directed through the pupil by a bi-convex lens, while the structures thus strongly illuminated may be inspected by the aid of another. The ophthalmoscopic mirror displays any opacities in the lens as dark spots or lines, by which the return of light from the fundus is intercepted; the focal illumination displays them in their natural colours, as brownish or greyish irregular clouds or striæ, which often commence in the periphery of the lens behind the iris, and encroach more or less upon the pupillary area. With the most ordinary care, the question of the presence of commencing cataract cannot be for a single instant in doubt; although I once met with a case in which a hasty observer had been led into error by some slightly misdirected eyelashes, which appeared as dark lines across the ophthalmoscopically illuminated pupil, and were mistaken for the striæ of lenticular opacity.

At the time when the ophthalmoscope was introduced into practice, the method of operating for senile cataract was that devised by Daviel, which had held its ground for more than a century. His original knife had been modified by Beer, and was a miniature form of the implement which is still used for cutting trusses of hay out of a rick. The blade was a right-angled triangle, with a cutting edge on the hypothenuse, which formed the point by its junction with the longer of the two sides containing the right-angle. From the point backwards, the blade increased in thickness as well as in width, the object of the increase being that it should completely fill its incision as it advanced, and should prevent any escape of the aqueous humour. The lower lid being controlled by an assistant, the surgeon raised the upper lid with his index finger, and steadied the eye by resting the middle finger against its inner portion near the caruncle. He then introduced the point of the knife into the cornea, near its margin and on the horizontal meridian, the cutting edge being usually upwards, carried the back of the blade straight across the anterior chamber to a corresponding point on the opposite side of the cornea, brought it out by counter-puncture, and completed his section by simply pushing straight on. In this way the upper half of the cornea was detached as a flap, but some operators preferred to make the flap downwards. The capsule of the lens was then opened by a pricker carried through the pupil, and the lens itself was pressed through the pupil, and ultimately through the corneal section. Any residual soft matter was removed as far as possible, and the eye was closed to await the development of the result.

